# Calcification Propensity (T50) Predicts a Rapid Decline of Renal Function in Kidney Transplant Recipients

**DOI:** 10.3390/jcm12123965

**Published:** 2023-06-10

**Authors:** Nathalie Hammer, David Legouis, Andreas Pasch, Aurélie Huber, Lama Al-Qusairi, Pierre-Yves Martin, Sophie de Seigneux, Lena Berchtold

**Affiliations:** 1 Service of Nephrology, Inselspital, 3010 Bern, Switzerland; nathalie.hammer@insel.ch; 2 Division of Intensive Care, University Hospital of Geneva, 1205 Geneva, Switzerland; 3 Calciscon AG, 2503 Biel, Switzerland; 4 Department of Physiology and Pathophysiology, Johannes Kepler University, 4040 Linz, Austria; 5 Service of Internal Medicine, Hospital La Chaux-de-Fonds, 2000 Neuchatel, Switzerland; 6 Division of Nephrology, Johns Hopkins University School of Medicine, Baltimore, MD 21218, USA; 7 Service of Nephrology, Department of Internal Medicine Specialties, University Hospital of Geneva, 1205 Geneva, Switzerland

**Keywords:** kidney transplant, decline of renal function, prediction, phosphocalcic markers

## Abstract

Background: Serum creatinine level, proteinuria, and interstitial fibrosis are predictive of renal prognosis. Fractional excretion of phosphate (FEP)/FGF23 ratio, tubular reabsorption of phosphate (TRP), serum calcification propensity (T50), and Klotho’s serum level are emerging as determinants of poor kidney outcomes in CKD patients. We aimed at analysing the use of FGF23, FEP/FGF23, TRP, T50, and Klotho in predicting the rapid decline of renal function in kidney allograft recipients. Methods: We included 103 kidney allograft recipients in a retrospective study with a prospective follow-up of 4 years. We analysed the predictive values of FGF23, FEP/FGF23, TRP, T50, and Klotho for a rapid decline of renal function defined as a drop of eGFR > 30%. Results: During a follow-up of 4 years, 23 patients displayed a rapid decline of renal function. Tertile of FGF23 (*p* value = 0.17), FEP/FGF23 (*p* value = 0.78), TRP (*p* value = 0.62) and Klotho (*p* value = 0.31) were not associated with an increased risk of rapid decline of renal function in kidney transplant recipients. The lower tertile of T50 was significantly associated with eGFR decline >30% with a hazard ratio of 3.86 (*p* = 0.048) and remained significant in multivariable analysis. Conclusion: T50 showed a strong association with a rapid decline of renal function in kidney allograft patients. This study underlines its role as an independent biomarker of loss of kidney function. We found no association between other phosphocalcic markers, such as FGF23, FEP/FGF23, TRP and Klotho, with a rapid decline of renal function in kidney allograft recipients.

## 1. Introduction 

Kidney transplantation is the best treatment mode for patients with end-stage kidney disease [1]. Glomerular Filtration Rate (GFR) normally stabilizes at approximately 60% of donor renal function before presenting a gradual decline influenced by numerous variables as drug toxicity, rejection episodes and infections [2,3]. Serum creatinine, proteinuria, and interstitial fibrosis are well-known predictors of kidney function evolution [4]. Defining novel non-invasive markers that could precisely predict the individual eGFR decline in kidney allograft recipients (KARs) is important in patient management. Amongst potential tools, mineral metabolism biomarkers are of interest as disorders in phosphorus and calcium homeostasis are common.

Klotho is a protein mainly expressed in kidney proximal and distal tubular cells. During the early phases of experimental chronic kidney disease (CKD), Klotho expression is decreased [5,6]. Lower Klotho serum levels are associated with a higher prevalence of cardiovascular disease, arterial stiffness and vascular calcification in the experimental setting and some clinical observations [7,8,9]. Low serum Klotho levels are also significantly associated with an increased risk of poor kidney outcomes in CKD, dialysed or transplanted patients [10]. Despite the unknown effect of Klotho in kidney allografts, its protective role in renal tubular cells and inhibition of renal fibrosis seems to impact the post-transplant ischemia-reperfusion injury and eventually alleviate delayed graft function [11,12]. 

FGF23 is a key phosphaturic hormone produced by osteocytes and osteoblasts, which increases early in CKD. FGF23 appears to be a sensitive marker of kidney disease and cardiovascular complications in the CKD population [13]. The phosphaturic action of FGF23 is based on its capacity to suppress renal phosphate reabsorption at the proximal tubules and thus to increase its urinary excretion at each nephron, referred as the fractional excretion of phosphate (FEP). Tubular reabsorption of phosphate (TRP), defined as 1-FEP, emerges as a possible surrogate marker for phosphate regulation in pre-dialysis CKD patients, as it correlates with renal function [14,15]. With the postulation that excreting phosphate is a marker of nephron stress, Bellasi et al. showed in a retrospective study that FEP is associated with end stage renal disease (ESRD), but not with all-cause mortality risk in a large cohort of stages 3b to 5 CKD patients [16]. Yamada and Kuro-o proposed the FEP to FGF23 ratio as an index that theoretically represents the number of healthy nephrons [17]. This ratio is an independent risk factor for renal progression [18] and was shown to be associated with aortic calcification in CKD [19]. Moreover, FGF23 is not only a risk marker for CKD progression and cardiovascular mortality in primary CKD but seems also important in KARs [13,20,21,22,23]. Elevated c-terminal FGF23 (cFGF23) concentration was associated with overall graft loss in a previous observation [24].

Kidney transplantation incompletely mitigates the cardiovascular risk despite restoring renal function. KARs have markedly accelerated vascular calcification even with a stable renal function [25,26,27]. Serum calcification propensity test (T50) was developed to monitor the maturation time of calciprotein particles in serum [28]. High calcification propensity (or low T50) is closely associated with progressive aortic stiffening and increased long-term mortality in CKD patients [29]. Conversely, a prolonged T50 indicates a high residual capacity of serum in the patient, which prevents the formation of secondary calciprotein particles and is, therefore, indicative of an intact endogenous defence against calcification. T50 was shown as an independent determinant of graft failure in kidney transplant recipients [30,31]. Whether T50 is associated with rapid decline of renal function in KARs is unknown.

Altogether, mineral metabolism markers may be interesting as non-invasive prognosis markers of renal function loss in KARs. In this study, we aim to analyse the association between FGF23, FEP/FGF23, TRP, T50, and Klotho with the rapid decline of renal function in KARs, defined as a loss of eGFR > 30% at 4-years follow-up.

## 2. Material and Methods

**Figure 1:**
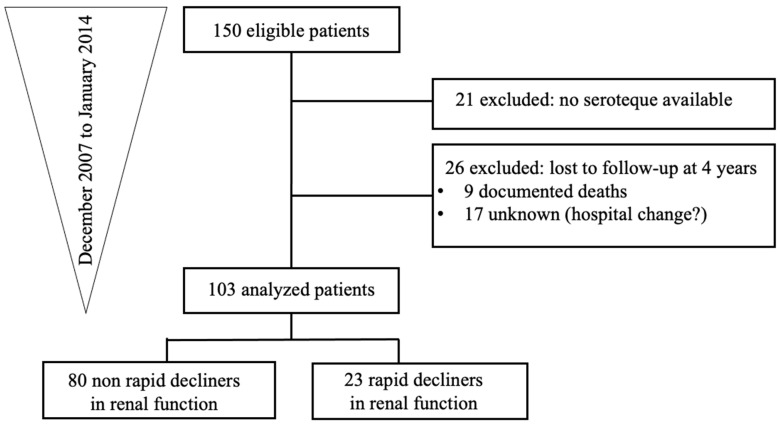
Flow chart.

We designed a retrospective study including adult KARs in whom serum was kept for clinical reasons. Patients aged 18 or older, who received a kidney transplant between 1982 and 2013, who were followed routinely and whose serum was collected in 2015 at our hospital, were eligible for enrolment. Two nephrologists, not in charge of the patients, randomly selected 150 patients between January 2007 to December 2014. We excluded 21 patients because of the lack of available serum samples and 26 patients because of the lack of available follow-up, leaving 103 patients for the current analysis (Figure 1). 

Baseline characteristics, including medical history, co-morbidities and transplantation-related outcomes were collected through patient records. The patient’s blood pressure, weight and size were measured routinely during follow-up visits. Serum creatinine and other standard laboratory values were measured during routine follow-up visits or hospitalizations and recorded at the time of the collected sample. Standard biochemical analyses were performed in our hospital using the routine automated analysers. eGFR was calculated using the Chronic Kidney Disease Epidemiology Collaboration equation from 2012 (CKD-EPI 2012) [32]. Creatinine was measured using IDMS-traceable Jaffe’s kinetic method. 

We defined a threshold value for the rapid decline of eGFR as a decrease higher than 30% at 4-years follow-up, as already described in previous studies [33,34,35]. Frozen samples were used to measure FGF23, Klotho and T50 in batch. Serum FGF23 levels were measured by the ELISA system using the C-TER Immunotopics kit [36]. An ELISA essay kit from IBL was used to measure serum levels of soluble Klotho [37]. The serum calcification propensity test (T50) was performed using a Nephelostar nephelometer [28]. Fractional excretion of phosphate (FEP) was calculated using the following formula: FEP = (24 h urine phosphate × serum creatinine)/(serum phosphate × 24 h urine creatinine) × 100. TRP was calculated as 1-FEP. TmP/GFR calculation depends on TRP: if TRP is ≤ 0.86, TmP/GFR = TRP × serum phosphate. If TRP > 0.86, TmP/GFR is defined as ((0.3 × TRP)/(1 − (0.8 × TRP)) × serum phosphate. Technicians from the laboratories were blinded to the clinical data and other results. The study was approved by the local ethical committee for human studies (CER 14-149) and performed according to the Declaration of Helsinki principles. All the patients were contacted to provide written informed consent to participate in this retrospective study. 

### 2.1. Statistical Analysis

Continuous variables are expressed as mean +/− SD and categorical variables are expressed as numbers and percentages. *p*-values were calculated with Student’s *t*-test for continuous variables or Chi^2^ for categorical variables. FGF23 and proteinuria were logarithmically transformed before analysis due to abnormal distribution. The statistical significance was determined as a *p* < 0.05, and all tests were two-tailed. For simple correlation analyses between phosphocalcic parameters, including T50 and renal function, we performed Pearson’s tests after controlling the linear associations with scatter plots. 

To test the hypothesis that phosphocalcic markers could predict a rapid decline of renal function, we performed log-rank tests for trends, comparing for each variable the risk of renal function loss. Time-to-event data were evaluated using Kaplan–Meier estimates and Cox proportional-hazard models. Hazard ratios, 95% confidence intervals and *p* values were calculated. Proportionality of hazard was graphically verified by plotting log minus log of survival against time. Statistical analyses were performed using STATA 16.1 (StataCorp, College Station, TX, USA).

### 2.2. Outcomes

The primary outcome was the rapid decline of renal function, defined as a decline of eGFR of >30% over 4 years. For participants who died or were lost to follow-up, the last available eGFR was used to assess the primary outcome.

## 3. Results

### 3.1. Characteristics of the Study Population

We included 150 KARs at different time points from transplantation (Figure 1). Among them, only 103 were analysed because 21 patients had no serum available and 26 patients were lost to follow-up at 4 years. The mean age was 56 years old, with mainly male gender (63%) and Caucasian origin (98%). The clinical characteristics of the study participants are shown in Table 1. Demographics data from the 2 groups are comparable. In total, 23 patients were defined as having a rapid decline in renal function, defined as >30% of eGFR decline. 

### 3.2. Association between Phosphocalcic Biomarkers and Renal Function at Baseline 

**Figure 2:**
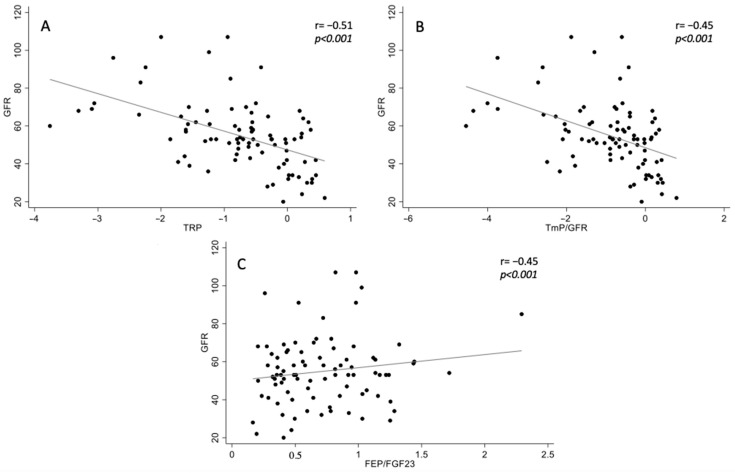
Correlations between TRP, TmP/GFR, FEP/FGF23 and renal function defined by eGFR. Scatter plots of TRP (**A**), TmP/GFR (**B**) and FEP/FGF23 (**C**) versus GFR. Each symbol represents one patient. The continuous line indicates least-square linear regression. The correlation coefficient (r) and significance (*p*) are displayed in each scatter plot.

At baseline, phosphate (r = −0.19, *p* = 0.048), Klotho (r = 0.27, *p* = 0.006), logFGF23 (r = −0.47, *p* < 0.001), bicarbonate (r = 0.25, *p* = 0.01) correlated with eGFR as we previously described. TRP (r = −0.51, *p* < 0.001) (Figure 2A), TmP/GFR (r = −0.4, *p* < 0.001) (Figure 2B) were also associated with renal function [4]. FEP/FGF23 (r = 0.15, *p* = 0.17) (Figure 2C) did not correlate with renal function.

### 3.3. Univariable and Multivariable Analysis of Predictors of Renal Function Decline 

During a follow-up of 4 years, 23 patients (23/103 = 22.3%) displayed a rapid decline of renal function according to our definition. Baseline mean eGFR was 54.4 ± 19.9 mL/min/1.73 m^2^ in the group with rapid decline of renal function and 56 ± 18.4 mL/min/1.73 m^2^ in the non-rapid decline of renal function group. 

We tested the known risk factors of a rapid decline of renal function used in the kidney failure risk calculation: age, sex, eGFR, albuminuria, albumin, phosphate, bicarbonate, and calcium at baseline. Sex, albumin, phosphate, bicarbonate, and calcium were not associated with a rapid decline of renal function in our population (Supplementary Table S1 and Figure S1). Albuminuria > 300 mg/24 h was strongly associated (HR 4.37, 95% CI [1.46–13.3], *p* value = 0.008) with a rapid decline of renal function (Supplementary Table S1 and Figure S1G). 

Tertiles of Klotho, FGF23, FEP, FEP/FGF23 ratio, TRP and TmP/GFR were not associated with a rapid decline of renal function. However, the first tertile of T50 was strongly associated with a rapid decline of renal function in kidney allograft patients (HR 4.26, 95% CI [1.2–15.1], *p* = 0.025) (Table 2).

**Figure 3:**
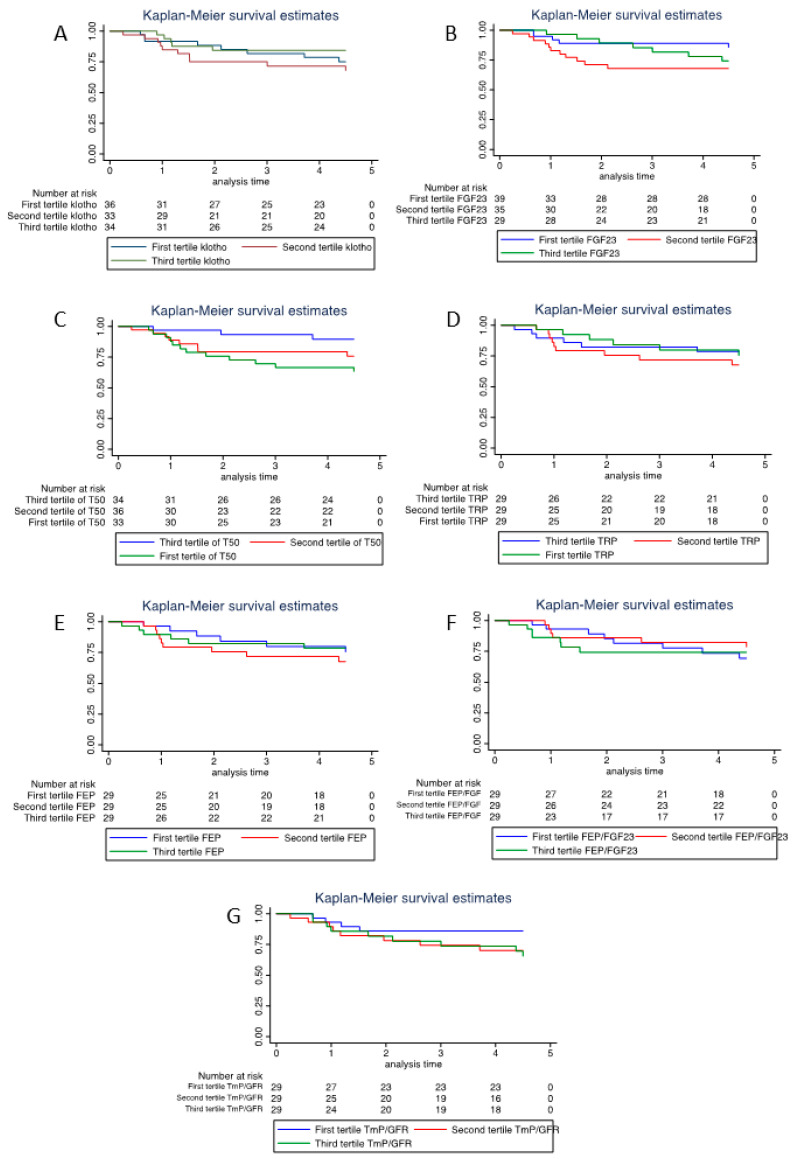
Kaplan–Meier curves of the rapid decline of renal function by (**A**) tertile of Klotho, (**B**) tertile of FGF23, (**C**) tertile of T50, (**D**) tertile of TRP, (**E**) tertile of FEP, (**F**) tertile of FEP/FGF23 and (**G**) tertile of TmP/GFR.

For clarity in graphs and the interpretation of results, we decided to use the higher values of the third T50 tertile as a reference and the lower as a first tertile. The same reflexion applies to tertile TmP/GFR. Kaplan Meier curves are shown in Figure 3. 

### 3.4. Multivariable Analysis of Predictors of Renal Function Decline 

In the multivariable analysis, including eGFR at baseline, albuminuria and T50, eGFR < 30 mL/min, albuminuria >300 mg/day and lower T50 were associated with a rapid decline of renal function (Table 3).

## 4. Discussion 

In this retrospective study including 103 kidney allograft recipients followed up to 4 years, phosphocalcic biomarkers such as FGF23, Klotho, FEP/FGF23 ratio and TRP were not associated with a rapid decline of renal function. T50 was in contrast associated with a rapid decline of renal function and this association remained significant in multivariable cox analysis including albuminuria and eGFR at baseline. 

A shortened T50 reflects an abnormal mineral metabolism that predisposes to vascular vessels calcification leading to fatal or nonfatal cardiovascular disease in ESRD patients [38]. A high arterial calcification burden increases arterial stiffness and is associated with a faster decline of kidney function in patients with arterial hypertension and/or CKD. Although the underlying mechanisms are not completely understood, Pruijm et al. found that a shortened T50 was associated with lower renal tissue oxygenation (confirmed by MRI) and perfusion in hypertensive and CKD patients [39]. As restoration of renal function after transplantation does not mitigate cardiovascular risk due to accelerated vascular calcification in the transplanted patient, such alterations of perfusion may also occur in KARs. Since inflammation may occur for various reasons, and pro-inflammatory cytokines restrict the synthesis of Fetuin-A in the liver, the ability to counteract the calcium mineral disbalance-driven injury progression is further reduced [40]. This may lead to alterations of T50 and progression of vasculopathy in the kidney. Consequently, accumulating calciprotein particles might subsequently affect graft function by ischemia, leading to a more rapid decline of renal function. Further studies are needed to evaluate prospectively the impact of T50 measurement on KARs prognosis and care. 

In a study including stable kidney transplant recipients with a median transplant vintage of 3.9 years, patients with lower T50 had a 2.3-fold increased risk of cardiovascular disease. Even after multivariate adjustment, each standard deviation decrease in T50 was independently associated with a 22% greater CVD risk [41]. While low T50 values are shown to be independently associated with increased all-cause mortality (HR 1.43, 95% CI [1.11–1.85]; *p* = 0.006), cardiovascular mortality (HR 1.55, 95% CI [1.04–2.29], *p* = 0.03), and risk of graft dysfunction (HR, 3.80; 95% CI [1.53–9.45], *p* = 0.004) in an independent cohort [31], this is the first time that T50 emerges as a new marker of renal function decline in KARs. This marker may thus be of interest to determine renal prognosis and as a potential future therapeutic target.

We did not observe any association between Klotho level after transplantation and the rapid decline of renal function at 4 years. The inclusion of patients after transplantation may explain those results. Indeed, the pre-transplant Klotho level seems to be decisive for the post-transplant period, independent of transplant or donor characteristics [42]. Deceased donor Klotho polymorphism, also predicts early transplant glomerular lesions and function [43]. While Klotho levels of the recipient and donor before transplantation may be significant, Klotho levels measured after transplantation are not associated with decline of renal function in our cohort.

No association between FGF23 and the rapid decline of renal function in KARs was observed. Up to now, few studies have evaluated the role of FGF-23 in mortality and graft loss prediction in KARs, and their results are contradictory. In recent studies including patients with a median transplantation vintage of about 6–7 years, FGF23 was an independent risk factor for allograft loss, cardiovascular, and all-cause mortality [22,44]. In contrast, FGF23 was not an independent risk factor for mortality in the short period (until 48 months) post-transplant in a French cohort [45]. Thus, FGF23 is not a clear marker for renal function decline in KARs.

TRP was also not associated with a rapid decline of renal function in kidney allograft patients despite previous promising studies.

The major limitation of our study is its monocentric, retrospective and cross-sectional design. A sample size calculation was made at the time of the research protocol and the risk of a negative study due to a lack of power was low. Some of our negative results might nevertheless be explained by the small sample size. Technical limitations in regard to Klotho measurement in human research may also have impacted results interpretation [37]. In addition, Klotho serum levels in KARs may not reflect renal expression. The same limitation applies to FGF23 measurement. In our study, we measured the C-terminal portion of FGF23 (cFGF23). We therefore cannot completely exclude that intact FGF23 (iFGF23) would have a different predictive value over the C-terminal portion measurement, although it has been described that cFGF23 concentrations have a better discriminatory ability than iFGF23 concentration in predicting overall (all-cause) graft loss [24].

## 5. Conclusions

In this retrospective study, T50 was the only parameter associated with a rapid decline of renal function in kidney allograft patients. T50 is a well-described predictive marker for its reflection of intravascular calcification and risk of cardiovascular mortality. We confirm that this marker is predictive of renal function evolution in KARs, but we were not able to demonstrate an association between FGF23, Klotho, FEP/FGF23 and TRP and the rapid decline of renal function. Our study confirms the need to initiate large-scale, prospective, multicentre studies for longitudinal follow-up of KARs and to integrate T50 as a marker of renal prognosis in this population.

## 6. Three Statements

(1). What is known:

a. Serum creatinine and proteinuria are predictive of renal prognosis.

b. Novel individualized non-invasive markers of renal function decline are needed in kidney allograft recipients (KARs).

(2). What this study adds:

a. This study tries to define mineral metabolism markers that could precisely predict the individual eGFR decline in KARs.

b. T50 is associated with a rapid decline of renal function

(3). What impact this may have on practice:

a. Finding new non-invasive predictors of renal function decline in KARs could help clinicians to monitor and anticipate kidney allograft dysfunction, and thus prevent invasive procedures such as kidney biopsy.

b. T50 may be used to predict a rapid decline of renal function. However, other mineral metabolism alterations may not play the same role in KARs’ renal prognosis as in native kidney patients.

## Figures and Tables

**Table 1:** Baseline characteristics of the study population.

	Total (n = 103)	eGFR Decline at 4 Years ≤ 30% (n = 80)	eGFR Decline at 4 Years > 30% (n = 23)	*p* Value
Characteristics	-			
Age, years	56 ± 14	56 ± 14	57 ± 13	0.57
Male, n (%)	63 (61.2)	48 (60)	15 (65.2)	0.65
Caucasian, n (%)	98 (95.2)	76 (95)	22 (95.7)	0.89
Clinical parameters				
Systolic blood pressure (mmHg) *a	132 ± 16	132 ± 15	131 ± 20	0.76
Diastolic blood pressure (mmHg) *a	80 ± 12	80 ± 12	78 ± 12	0.44
BMI (kg/m^2^)	25.3 ± 4.3	25.4 ± 4.3	24.8 ± 4.4	0.57
Dialysis before tx, n (%) *c	40 (72.7)	31 (73.8)	9 (69.2)	0.75
Dialysis duration, mths, *c	24.2 ± 29.4	24.45 ± 30.8	23.3 ± 25.6	0.90
Donor age, years, *c	53 ± 14	54 ± 14	54 ± 15	0.94
Living donor transplant, n (%)	40 (38.8)	32 (40)	8 (34.8)	0.65
Graft vintage (year)	9.48 ± 7.3	9.45 ± 7.1	9.6 ± 8.2	0.94
2nd or 3rd transplant, n (%)	14 (13.6)	11 (13.8)	3 (13)	0.93
Rejection episodes, n (%)	46 (44.7)	32 (40)	14 (60.9)	0.08
Infections, n (%)	52 (66.7)	40 (66.7)	12 (66.7)	1
Opportunistic inf, n (%) *c				0.76
None	41 (51.3)	30 (49.2)	11 (57.9)	
1	31 (38.8)	25 (41)	6 (31.6)	
2	8 (10)	6 (9.8)	2 (10.5)	
NODAT, n (%)	11 (10.7)	9 (11.3)	2 (8.7)	0.73
Etiology of kidney disease, n (%)				
Diabetes	4 (3.9)	3 (3.8)	1 (4.4)	0.89
Hypertension	20 (19.4)	13 (16.3)	7 (30.4)	0.13
Glomerulonephritis	22 (21.4)	18 (22.5)	4 (17.4)	0.6
Polycystic kidney disease	20 (19.4)	15 (18.8)	5 (21.7)	0.75
Others (tubulo-interstitial nephritis…)	41 (39.8)	34 (42.5)	7 (30.4)	0.3
Treatment, n (%)				
CNI	92 (89.3)	77 (87.5)	22 (95.7)	0.27
Mycophenolate mofetil	80 (77.7)	61 (76.3)	19 (82.6)	0.52
Corticostéroids	60 (58.3)	43 (53.8)	17 (73.9)	0.08
ACE-I/ARB	54 (52.4)	39 (48.8)	15 (65.2)	0.16
Calcium channel blockers	50 (48.5)	37 (46.3)	13 (56.5)	0.39
Diuretics	7 (6.8)	4 (5)	3 (13)	0.18
Beta-blockers	56 (54.4)	44 (55)	12 (52.2)	0.81
Calcium	69 (67)	54 (67.5)	15 (65.2)	0.84
Vitamine D	88 (85.4)	68 (85)	20 (87)	0.82
Laboratory				
eGFR at baseline (mL/min/1.73 m^2^)	55.6 ± 18.6	56 ± 18.4	54.4 ± 19.9	0.72
eGFR at 4 years (mL/min/1.73 m^2^)	48.3 ± 19.7	53.9 ± 16.9	28.8 ± 16.1	<0.001
Proteinuria (g/d) *b	0.4 ± 1.4	0.4 ± 1.6	0.2 ± 0.2	0.5
Alb/Creat ratio (mg/d) *b	148.8 ± 283	116.5 ± 272.1	255.6 ± 298	0.04
Hemoglobine (g/L)	129 ± 14	129.8 ± 13.4	126.4 ± 15.9	0.31
Calcium (mmol/L)	2.41 ± 0.14	2.41 ± 0.13	2.42 ± 0.17	0.9
Phosphate (mmol/L)	1.09 ± 0.23	1.07 ± 0.2	1.13 ± 0.32	0.32
Vitamine D (nmol/L) *a	68.6 ± 21.6	68.7 ± 21.6	68.3 ± 22.1	0.94
Parathormone (pmol/L) *a	9.14 ± 4.6	8.8 ± 4.1	10.1 ± 6.1	0.25
Bicarbonate (mmol/L) *a	24.3 ± 3.4	24.3 ± 3.6	24.3 ±2.6	0.99
Albumin (g/L)	37.4 ± 3.6	37.7 ± 3.6	36.5 ± 3.4	0.18
FEP/FGF23 § ratio *b	0.72 ± 0.39	0.73 ± 0.4	0.7 ± 0.4	0.77
FEP (%)	26.2 ± 11.1	25.9 ± 10.3	27.1 ± 13.5	0.68
FGF23 (pg/mL)	43.6 ± 28.7	43.2 ± 29.7	45.2 ± 25.5	0.77
TRP ‡,*b	0.98 ± 0.01	0.98 ± 0	0.98 ± 0.01	0.68
TmP/GFR ¶ (mmol/L) *b	1.48 ± 0.3	1.45 ± 0.3	1.58 ± 0.5	0.12
Klotho (pg/mL)	734.9 ± 244.2	751.6 ±264	676.6 ± 147.4	0.2
T50 (min)	285.2 ± 56.7	291.9 ± 55.9	262 ± 54.6	0.03

Continuous variables are expressed as mean ± SD. Categorical variables are expressed as numbers and percentages; * Missing values: *a: 0–5; *b: 5–10; *c: >10; § FEP: fractional excretion of phosphate; FGF-23: c-terminal fibroblast growth factor 23; ‡ TRP: phosphate tubular reabsorption (plasma creatinine in umol/L); ¶ TmP/GFR: maximal tubular phosphate reabsorption; glomerular filtration rate; BMI body mass index, CNI calcineurin inhibitors, NODAT new onset diabetes after transplantation, ACE Inhibitors/ARB angiotensin-converting enzyme inhibitors, angiotensin receptor blockers, eGFR estimated glomerular filtration rate, Alb/Creat ratio albumin/creatinine ratio, T50 serum calcification propensity.

**Table 2:** Predictors of decline of renal function by univariable analysis.

	HR	95% CI	*p* Value
Tertile Klotho			
First tertile	Reference		
Second tertile	1.41	0.56–3.58	0.47
Third tertile	0.63	0.21–1.92	0.42
Tertile FGF23			
First tertile	Reference		
Second tertile	2.65	0.92–7.64	0.07
Third tertile	1.72	0.55–5.42	0.35
Tertile FEP			
First tertile	Reference		
Second tertile	1.52	0.54–4.28	0.43
Third tertile	0.97	0.31–3.02	0.96
Tertile FEP/FGF23			
First tertile	Reference		
Second tertile	0.72	0.25–2.08	0.54
Third tertile	1.01	0.37–2.8	0.98
Tertile T50			
Third tertile	Reference		
Second tertile	2.78	0.74–10.48	0.13
First tertile	4.26	1.2–15.09	0.025
Tertile TRP			
First tertile	Reference		
Second tertile	1.56	0.56–4.4	0.4
Third tertile	1.03	0.33–3.19	0.96
Tertile TmP/GFR			
Third tertile	Reference		
Second tertile	2.29	0.69–7.62	0.18
First tertile	2.53	0.78–8.22	0.12

**Table 3:** Predictors of decline of renal function by multivariable analysis.

	HR	95% CI	*p* Value
eGFR			
≥45 & <60 mL/min/1.73 m^2^	0.27	0.05–1.51	0.14
<45 & ≥30 mL/min /1.73 m^2^	0.28	0.06–1.33	0.11
<30 mL/min/1.73 m^2^	0.1	0.01–0.97	0.047
Albuminuria			
30–300 mg/24 h	1.89	0.65–5.48	0.24
>300 mg/24 h	4.47	1.39–14.52	0.012
Tertile T50			
Second tertile	2.5	0.62–10.01	0.2
First tertile	3.86	1.01–14.7	0.048

## Data Availability

The data presented in this study are available on request from the corresponding author.

## Supplementary Materials

The following supporting information can be downloaded at: https://www.mdpi.com/article/10.3390/jcm12123965/s1, Figure S1: Kaplan-Meier curves of a rapid decline of renal function by (**A**) sex, (**B**) calcium, (**C**) phosphate, (**D**) bicarbonate, (**E**) GFR, (**F**) albuminemia and (**G**) albuminuria; Table S1: Predictors of decline of renal function by univariable analysis.
