# Supplementary material for: Calcification Propensity (T50) Predicts a Rapid Decline of Renal Function in Kidney Transplant Recipients
Source: J Clin Med. 2023 Jun 10;12(12):3965. doi: 10.3390/jcm12123965 (PMC10298924; doi:10.3390/jcm12123965)
Supplement: Supplementary file 1 [file jcm-12-03965-s001.zip › jcm-2436306-supplementary.pdf]

Journal of
Clinical Medicine

MDPI

Table S1. Predictors of decline of renal function by univariable analysis.

	HR	95% CI	*p* Value
Age (per 10 years)	1.01	0.98–1.04	0.54
Sex			
Male	Reference		
Female	1.14	0.48–2.68	0.77
eGFR			
>60 mL/min/1.73 m²	*Reference*		
≥45 & < 60 mL/min/1.73 m²	0.41	0.08–2.11	0.28
<45 & ≥30 mL/min/1.73 m²	0.53	0.12–2.33	0.4
<30 mL/min/1.73 m²	0.39	0.04–4.32	0.44
Albumin			
≥35 mmol/l	Reference		
<35 mmol/l	0.99	0.37–2.69	0.99
Phosphate			
≤1.45 mmol/l	Reference		
>1.45 mmol/l	2.27	0.53–9.68	0.27
Calcium			
≥2.2 mmol/l	Reference		
<2.2 mmol/l	2.07	0.49–8.83	0.33
Bicarbonate			
≥22 mmol/l	Reference		
<22 mmol/l	0.58	0.13–2.45	0.46
Albuminuria			
<30 mg/24 h	Reference		
30–300 mg/24 h	1.55	0.56–4.27	0.85
>300 mg/24 h	4.37	1.46–13.03	0.008

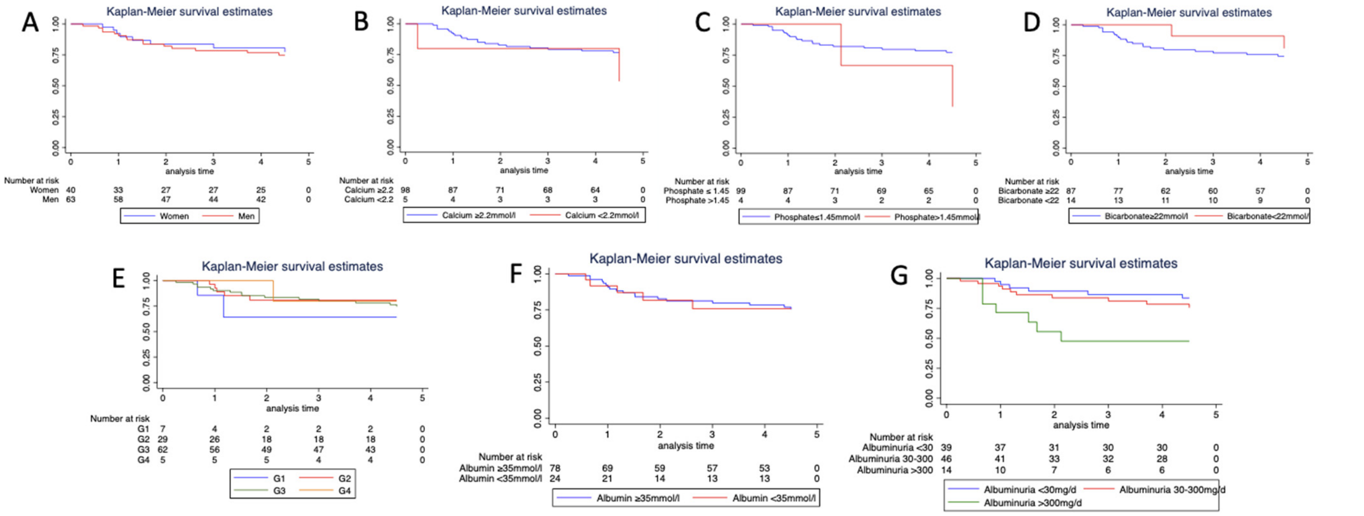

Figure S1. Kaplan-Meier curves of a rapid decline of renal function by (**A**) sex, (**B**) calcium, (**C**) phosphate, (**D**) bicarbonate, (**E**) GFR, (**F**) albuminemia and (**G**) albuminuria.
